# Potato-Resistant Starch Supplementation Improves Microbiota Dysbiosis, Inflammation, and Gut–Brain Signaling in High Fat-Fed Rats

**DOI:** 10.3390/nu11112710

**Published:** 2019-11-08

**Authors:** Elizabeth A. Klingbeil, Carolina Cawthon, Rebecca Kirkland, Claire B. de La Serre

**Affiliations:** Department of Foods and Nutrition, University of Georgia, Athens, GA 30602, USAccawthon@uga.edu (C.C.); rkirklan@uga.edu (R.K.)

**Keywords:** gut microbiota, resistant starch, inflammation, glucose tolerance, vagal nerve, obesity

## Abstract

(1) High-fat (HF) diet leads to gut microbiota dysbiosis which is associated with systemic inflammation. Bacterial-driven inflammation is sufficient to alter vagally mediated satiety and induce hyperphagia. Promoting bacterial fermentation improves gastrointestinal (GI) epithelial barrier function and reduces inflammation. Resistant starch escape digestion and can be fermented by bacteria in the distal gut. Therefore, we hypothesized that potato RS supplementation in HF-fed rats would lead to compositional changes in microbiota composition associated with improved inflammatory status and vagal signaling. (2) Male Wistar rats (*n* = 8/group) were fed a low-fat chow (LF, 13% fat), HF (45% fat), or an isocaloric HF supplemented with 12% potato RS (HFRS) diet. (3) The HFRS-fed rats consumed significantly less energy than HF animals throughout the experiment. Systemic inflammation and glucose homeostasis were improved in the HFRS compared to HF rats. Cholecystokinin-induced satiety was abolished in HF-fed rats and restored in HFRS rats. HF feeding led to a significant decrease in positive c fiber staining in the brainstem which was averted by RS supplementation. (4) The RS supplementation prevented dysbiosis and systemic inflammation. Additionally, microbiota manipulation via dietary potato RS prevented HF-diet-induced reorganization of vagal afferent fibers, loss in CCK-induced satiety, and hyperphagia.

## 1. Introduction

Prevalence of obesity has soared to 93.3 million people in the United States [1]. Obesity has been characterized as a low-grade inflammatory state, and inflammation plays a critical role in both the exacerbation of obesity and the development of co-morbidities such as diabetes [2].

There is accumulating evidence that the chronic low-grade inflammation characteristic of obesity is at least partially controlled by the gut microbiota [3]. The gastrointestinal (GI) tract is home to over 10^14^ microorganisms, primarily bacteria, and microbiota makeup can influence host physiology and behavior [4]. Microbiota composition changes with diet and is especially responsive to/can be modulated by dietary fats [5,6], sugars [7], and fibers [5,8]. An abnormal microbiota composition, or dysbiosis, has been associated with increased adiposity in both humans [9,10] and animal models [7,11]. Obesity-associated microbiota is characterized by an increase in its pro-inflammatory potential [12,13] Additionally, dysbiosis alters GI epithelial barrier function, allowing bacterial by-product to exit the gut into the circulatory system [6,13,14]. Chronic administration of pro-inflammatory bacterial lipopolysaccharide (LPS) is sufficient to increase food intake [15] and induce weight gain [15,16] and insulin resistance [16], showing a direct relationship between bacterial products and development of obesity and associated comorbidities.

The GI microbiota composition can alter gut–brain communication, potentially affecting vagally-mediated post-ingestive feedback and intake regulation. Gut originating satiety signals, such as cholecystokinin (CCK), are released in response to feeding and act on vagal afferents to promote meal termination [17]. Animals fed high-fat (HF) or high-sugar diets displayed marked dysbiosis associated with reduction in isolectin B4 (IB4)-positive fibers at the level of the nucleus of solitary tract (NTS) where vagal afferents terminate [7]. Isolectin B4 binds to unmyelated c-fibers which, in the medial NTS, are predominantly of vagal origin [18]. Gut–brain vagal remodeling may be linked to bacterial-driven inflammation as antibiotic administration in HF-fed rats normalizes microbiota composition, NTS IB4 staining, and the accompanying immune cells activation observed in the NTS [19].

In addition, to affect energy homeostasis, chronic inflammation can promote the development of metabolic disorders, especially insulin resistance. Pro-inflammatory cytokines, such as tumor necrosis factor (TNF)-α and interleukin (IL)-1β, can promote insulin receptor substrate-1 (IRS1) phosphorylation at serine 307 (p-IRS1, Ser307), inhibiting insulin action [20,21].

Therefore, preventing dysbiosis and/or preserving the gut epithelial barrier integrity may inhibit systemic inflammation and prevent weight gain and insulin resistance.

Treatments with prebiotics [16] and antibiotics [22] can restore epithelial barrier function. In humans, prebiotic supplementation has been associated with an increase in markers of bacterial fermentation [23]. Short-chain fatty acids (SCFAs) are the main products of bacterial fermentation; acetate, propionate, and butyrate are the predominant SCFAs found in the intestine [24]. Short-chain fatty acids stimulate the production and differentiation of enterocytes, improving mucus production and epithelial health [25,26]. In animal models, supplementation with soluble fibers increases SCFA production, decreases inflammation, and positively affects glucose homeostasis [16,22,27].

Similar to fibers, resistant starch (RS) is a naturally occurring compound that escapes digestion in the proximal gut and can reach the distal GI (ileum and colon) and be fermented by gut microbiota [28,29]. Starch can resist digestion due to the fact of entrapment within a food (RS_1_), their chemical structure (RS_2_), or retrogradation during cooking (RS_3_) [28]. In humans, supplementation with RS has been shown to increase fecal SCFA [28]. Consumption of RS_2_ enhances acetate production, and RS_3_ drives propionate production [28]. There is evidence that RS consumption is associated with positive health outcomes: in piglets, supplementation with 10 g/day of potato improves insulin resistance [30], while in humans, corn RS consumption (24 g/d) is associated with lower fasting glucose levels [31]. Additionally, maize RS_2_ supplementation in rodents has been shown to alter gut microbiota composition [32] and improve inflammatory status [33].

Americans consume on average 4.9 g of RS daily [34]. Among commonly consumed foods, potatoes are a good source of RS, providing 2 to 5 g of RS per 100 g. The RS contents in potatoes vary with cooking method and temperature but are fairly constant among commonly consumed varieties of potatoes [35]. Raw potatoes are particularly rich in RS_2_; potato starch granules contain highly phosphorylated amylopectin and amylose that are not easily hydrolyzed [36]. Higher amounts of RS in raw potatoes have been shown to increase digestion time, potentially providing similar benefits as fermentable fibers [29].

There is limited knowledge on the potential protective effects of potato RS supplementation on gut microbiota composition, inflammatory status, and gut–brain signaling in diet-induced obesity models. In this study, we hypothesized that potato RS supplementation would prevent the onset of diet-driven microbiota dysbiosis, preserving gut–brain communication and preventing weight gain and metabolic dysfunctions associated with HF feeding.

## 2. Materials and Methods 

### 2.1. Animals and Diets

Twenty-four male Wistar rats were procured from Envigo (Indianapolis, IN, USA) and single-housed in wired-hanging cages in a temperature-controlled animal facility with a 12 h light–dark cycle. Following three days of habituation, animals were divided into three groups (*n* = 8 per group) and fed either a regular control chow diet (13% kcal from fat), HF diet (45% kcal from fat), or a HF diet supplemented with potato RS (HFRS) for 8 weeks. Animals were randomly assigned to groups and there were no differences in body weight at baseline among groups.

The chow control diet was ordered from Lab Supply (Fort Worth, Texas, PicoLab (5053), USA). The HF and HFRS diets were custom-made by Research Diets (New Brunswick, NJ, USA) and matched for energy density, macronutrient, and fiber contents (Table 1). The cornstarch and a portion of the maltodextrin in the 45% fat HF diet (D12541) were replaced with raw, unmodified potato starch in the HFRS diet (D17101605) (Bob’s Red Mill, Milwaukie, OR, WI, USA). Calculations were originally made based on the assumption that raw potato starch contains 50% RS [37]. Energy density for RS was calculated at 2.8 kcal/gram [38]; energy density for the 50% digestible portion of the potato starch was estimated at 4 kcal/gram. To make up for the lower overall energy density of the potato starch powder, maltodextrin contents were increased in the HFRS diet (Table 1). The HFRS diet was designed to contain 10% potato RS; this supplementation dose was based on previous data from our lab [27] and past research [39]. We verified the RS contents of the raw potato starch using a commercially available assay (Megazyme, Chicago, IL, USA) and determined that Bob’s Red Mill potato starch contains approximately 60% RS (Supplementary Tables S1 and S2), bringing our final supplementation level to approximately 12% and the HFRS energy density to 4.6 kcal/g (Table 1).

After 8 weeks on their respective diets, animals were fasted for 2 h and euthanized via CO_2_ inhalation. The sacrifice order was evenly distributed among groups and all tissues were collected within 6 h of light onset. Blood was sampled by cardiac puncture and rested on ice for 30 min before centrifugation at 4 °C at 8000 rpm for 10 min for serum collection. The GI tissues (i.e., duodenum, ileum, cecum, and feces) and visceral fat pad were collected, snap frozen, and stored at −80 °C. Brains were extracted and immediately placed in 4% paraformaldehyde solution (PFA) until brains sunk to the bottom of the tubes. Brains were then moved into 30% sucrose solution for cryoprotection. All animal care procedures were approved by the Institutional Animal Care and Use Committee of the University of Georgia, AUP A2017 08-017-R2.

### 2.2. Food Intake and Body Weight

Body weight and food intake were measured 3 days a week at the beginning of the light cycle for the entirety of the 8 week feeding intervention. Food intake (g) was determined by subtracting the amount of the remaining diets in the cages from the amount previously provided. Animals were housed in wired, hanging cages to ensure that food spillage was included.

### 2.3. Gut Microbiota and SCFA Quantification

Fecal pellets were collected at day 0 and after 8 weeks on the respective diets. Bacterial DNA were extracted from samples using the ZR Fecal DNA MiniPrep per the manufacturer’s protocol (Zymo Research, Irvine, CA, USA). The V4–V6 region of the 16S rRNA gene was amplified with the following primers: F515 (5′-GTGCCAGCMGCCGCGGTAA-3′) and RNextera (5′-CGACRRCCATGCANCACCT-3′) and targeted for sequencing by Ilumina MiSeq (University of Georgia Genomics Facility). Bacterial 16S sequences were processed with QIIME. The OTUs were picked based on 97% sequence similarity via the UCLUST algorithm. The OTUs were assigned to taxa through the Greengenes database. Chao index was calculated to determine α-diversity. The METAGENassist platform was used to assess β-diversity. Taxonomic abundances were log transformed for non-parametric tests. Multivariate analysis was conducted using the Galaxy platform linear discriminant analysis effect size (LEfSe) to identify taxonomic features discriminating of one or more groups.

The SCFAs in fecal samples were quantified using gas chromatography mass spectrometry at Mayo Clinic Laboratories (Rochester, MN, USA) as previously described [40].

### 2.4. GI Function

#### 2.4.1. GI Morphology and Goblet Cell Proliferation

Sections of distal small intestine (ileum) were cryosectioned (8 µm, Leica CM1900, Leica Biosystems, Wetzlar, Germany) and stained with alcian blue and nuclear fast red (Sigma–Aldrich, St. Louis, MO, USA). Villus length and the number of goblet cells (per crypt) were measured manually in well-oriented sections (5 measurements per ileal section) using a light microscope (BX40, Olympus) equipped with a digital camera (DP25, Olympus) and analysis software (DP2-BSW, Olympus).

#### 2.4.2. GI Permeability

Circulating LPS levels were measured as a proxy for intestinal barrier integrity. They were determined in serum using a pyrochrome lysate mix, a quantitative chromogenic reagent, diluted in Glucashield buffer which inhibits cross-reactivity with (1→3)-β-D-Glucans (Associates of Cape Cod, East Falmouth, MA, USA). Samples were diluted 1:10 in pyrogen-free water and heated for 10 min at 70 °C. Samples and reactive solution were incubated at 37 °C for 30 min, and absorbance was read at 405 nm on a Spectramax microplate reader (Molecular Devices, Sunnyvale, CA, USA).

#### 2.4.3. Real-Time PCR

The mRNA was extracted from ileum samples using the RNeasy Mini Kit (Qiagen, Valencia, CA, USA). The cDNA was synthesized using the RevertAid First Strand cDNA Synthesis Kit (Thermo Fisher Scientific, Franklin, MA, USA). The RT-PCR was run on a StepOnePlus real-time PCR system (Thermo Fisher Scientific) using SYBR Green PCR master mix (Thermo Fisher Scientific) and GLP-1 and Muc2 primers purchased from Integrated DNA Technologies. Data were analyzed according to the 2^−ΔΔCt^ method [41].

### 2.5. Sensitivity to Satiety Peptide CCK

After 6 weeks on their respective diets, animals were fasted for 12 h before receiving an administration of CCK (i.p., 0.22 nmL/kg, Bachem, Torrance, CA, USA) or saline (i.p., 400 µL, vehicle). A control experiment was conducted in which rats did not receive any injections. Food intake was measured 30, 60, and 120 min post-injection.

### 2.6. Glucose Homeostasis

#### Glucose Tolerances Test

After 7 weeks on respective diets, rats were fasted for 5 hours before oral gavage of 20% glucose solution (2 g/kg body weight). Glycemia was measured using a glucometer (Freestyle, Alameda, CA, USA) before oral gavage (0 min) and 15, 30, 60, 90, and 120 minutes after. Blood samples were collected at each time point and centrifuged as described above for insulin, glucagon, and GLP-1 analysis by multiplex ELISA (Meso Scale Diagnostics, Rockville, MD, USA).

### 2.7. Serum Inflammatory Markers

Serum collected at sacrifice was frozen at −80 °C and inflammatory markers were assessed using the FirePlex Rat Inflammation Immunoassay Panel (Abcam, Cambridge, MA, USA, ab235665) by the Abcam Fireplex Service Lab.

### 2.8. Immunohistochemistry

Hindbrains were cryosectioned (20 µm, Leica CM1900 from the caudal to the rostral region of the NTS (between bregma −14.16 and −12.93 mm). Sections were stained for IB4 (Novus Biologicals, Littleton, CO, USA) to visualize unmyelinated c-fibers and for ionized calcium binding adaptor molecule 1 (IBA1, Wako Chemicals, Richmond, VA, USA) which stains microglia.

After blocking with 10% goat serum in phosphate buffered saline (PBS), sections were incubated with primary antibody against IBA1 or with IB4 biotin conjugated overnight at 4 °C. Negative controls received 10% goat serum in PBS instead of primary antibody. Following washing, sections were incubated with secondary Goat anti-Rabbit IgG Alexa Fluor 488 conjugate (Invitrogen, Carlsbad, CA, USA) or ExtrAvidin-Cy3 (Sigma–Aldrich, St. Louis, MO, USA) for 1 h at 37 °C. Sections were mounted with fluoro gel (Electron Microscopy Sciences, Hartfield, PA, USA). Images of the NTS were captured at 20× magnification and stitched via a Nikon 80i imaging photomicroscope (Nikon, Tokyo, Japan) with a Nikon Digital Sight DS-Qi1Mc digital camera and filters for Alexa 488 and Extravidin-CY3. The IB4 and IBA1 fluorescence positive staining was quantified at the level of the area postrema as previously described [7] using binary imaging analysis based on principles described in Reference [42]. Analysis was completed using the Nikon Elements AR 3.0 Imaging software (Nikon).

### 2.9. Statistical Analysis

Unless stated otherwise (microbiota analysis), statistical analysis was performed using Prism software (Prism 6.0; GraphPad Software, La Jolla, CA, USA). Two-way repeated measures ANOVA was used to analyze body weight, energy intake, and OGTT with Tukey’s post-hoc test. One-way ANOVA was performed to analyze biochemical analyses. Non-normal datasets were analyzed with non-parametric methods. Differences were considered significant if *p* < 0.05. Data are presented as the mean ± SEM.

## 3. Results

### 3.1. Potato RS Reduces Weight Gain and Prevents Hyperphagia

**Figure 1:**
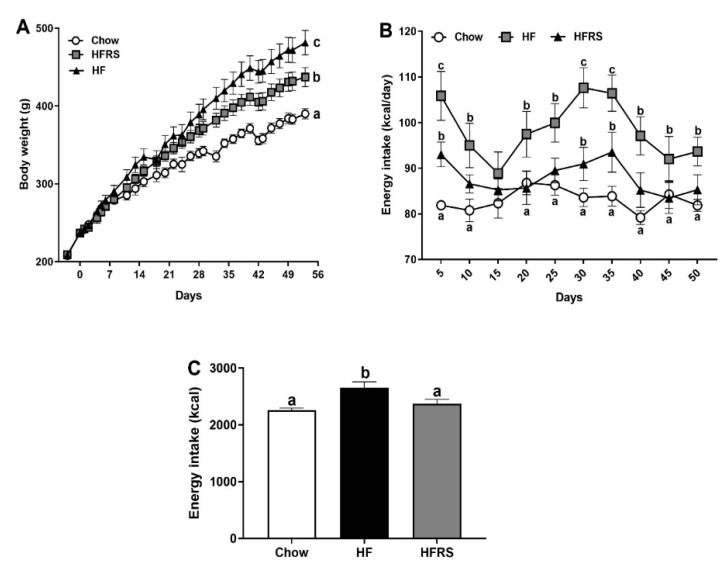
Potato RS reduces weight gain and prevents hyperphagia. (**A**) HF feeding led to a significant increase in body weight compared to control chow-fed conditions. The RS supplementation partially normalized weight gain. (**B**) The HF feeding led to a significant initial increase in energy intake in both HF and HFRS rats. The HF-fed rats’ intake was significantly higher than chow-fed animals throughout the study with the exception of week 2. After initial hyperphagia, HFRS rats’ energy intake normalized to the level of chow-fed rats, with the exception of week 4. (**C**) Overall energy intake was significantly higher in HF rats compared to both HFRS and chow control. Data are presented as the mean ± SEM; ^a, b, c^ different letters indicate statically significant (*p* < 0.05) differences among groups. HF = high fat, HFRS = high-fat resistant starch, *n* = 8 per group.

The HF fed animals gained significantly more weight than the chow control group over the course of the study (Figure 1A). They weighed significantly more starting at week 2 (chow 303.1 ± 6.8 g versus HF 334.7 ± 10.3 g, *p* < 0.05) and stayed heavier throughout the rest of the study. The RS supplementation led to a significant reduction in weight gain (HF 492.1 ± 16.2 g versus HFRS 445.1 ± 12.2 g, *p* < 0.01) but did not fully prevent a diet-induced increase in body weight as HFRS rats still weighed significantly more than the chow-fed control animals (chow 397.5 ± 6.7 g versus HFRS 445.1 ± 12.2 g, *p* < 0.01).

The HF-fed rats consumed more kcal than the chow control group for the entire duration of the study (Figure 1B) with the exception of week 2. The RS supplementation led to a significant decrease in diet-induced hyperphagia. The RS-supplemented rats initially displayed hyperphagia when first exposed to the diet (chow 82.6 ± 0.8 versus HFRS 93.76 ± 2.7 kcal/day, *p* < 0.001), although this first hyperphagic phase was significantly reduced compared to the HF group (HF 105.2 ± 5.3 versus HFRS 93.76 ± 2.7 kcal/day, *p* < 0.001). Throughout the rest of the experiment, the HFRS-fed rats’ intake was fairly similar to the chow-fed animals’ intake with the exception of week 4 (chow 84.3 ± 2.0 versus HFRS 91.5 ± 3.6 kcal/day, *p* < 0.05). Overall, over the 8-week period, HF rodents consumed significantly more kcal in total than either chow or HFRS rats (Figure 1C) (HF 2652 ± 295 versus chow 2260 ± 105 kcal, *p* < 0.01 and versus HFRS 2373 ± 216 kcal, *p* < 0.05). While chow and HFRS rats’ intake did not significantly differ (*p* = 0.56).

### 3.2. Potato RS Improves HF Diet-Driven Microbiota Dysbiosis

**Figure 2:**
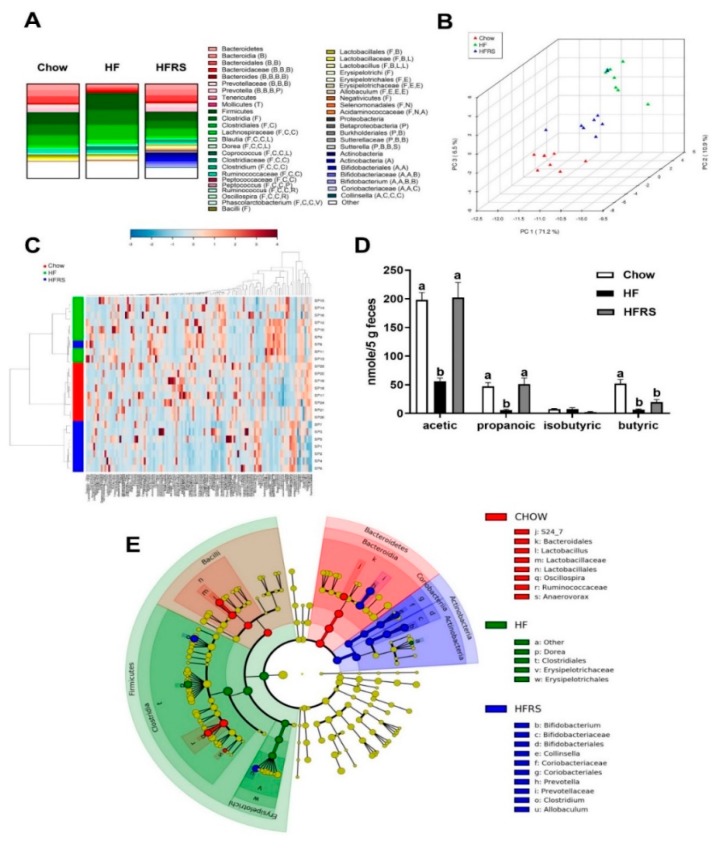
Potato RS improves microbiota dysbiosis. Microbiota abundance for chow, HF, and HFRS diet groups (**A**). The graph represents all abundances >1% at all phylogenic levels. Phyla: B: Bacteroidetes, F: Firmicutes, T: Tenericutes, P: Proteobacteria, A: Actinobacteria, V: Verrucomicrobia Class: B: Bacilli, B: Bacteroidia, C: Clostridia, E: Erysipelotrichia; M: Mollicutes, N: Negativicutes, D: Deferribacteres, A: Actinobacteria, V: Verrucomicrobiae, B: Betaproteobacteria. Order: B: Bacteroidales, C: Clostridiales, L: Lactobacillales, E: Erysipelotrichiales, B: Burkholderiales, B: Bifidobacteriales. Family: C: Clostridiaceae, L: Lachnospiraceae, R: Ruminococcaceae, L: Lactobacillaceae, E: Erysipelotrichaceae, D: Desulfovibrionaceae, B: Bifidobacteriaceae, V: Verrucomicrobiaceae, (A): Acidaminococcaceae. The PCA plot (run with all phylogenic levels, 121 normalized taxa abundances) shows similarities in overall microbiota profiles between chow and HFRS, while HF-fed rats displayed a distinct microbiota profile (**B**). The metagene heat-map displays microbiota characteristics among diet groups (**C**). RS supplementation developed a significantly different microbiota profile than HF. RS supplementation significantly improved fecal SCFA content (**D**). GALAXY cladogram highlights taxa characteristic of diet intervention, including *Bifidobacterium* bloom in HFRS (**E**). The LDA of 4.0 was used for the GALAXY cladogram. Data are presented as the mean ± SEM; ^a, b, c^ different letters indicate statically significant (*p* < 0.05) differences among groups. HF = high fat, HFRS = high-fat resistant starch, *n* = 8 per group.

The HF-fed rats displayed an overall dysbiotic microbiota profile that was significantly different from the chow-fed rats (Figure 2 and Supplementary Figure S1). The HF feeding led to marked changes in microbiota composition, characterized by an increase in the Firmicutes:Bacteriodetes ratio (chow 2:1 versus HF 8.6:1) and abundances of Clostridia (chow 54.28 ± 3.2 versus HF 76.18 ± 1.9, *p* < 0.0001). The increase in Clostridia abundances was driven by a bloom in families Dorea (*p* < 0.001) and Peptococcaceae (*p* < 0.001). The LEfSe showed that the Firmicutes classes Clostridia and Erysipelotrichi were characteristic of HF fed rats, in particular the order Clostridiales (Family, Dorea) and Erysipelotrichiales (Family, Erysipelotrichaceae) (Figure 2E).

Potato RS supplementation normalized the Firmicutes:Bacteriodetes ratio (HF 8.6:1 versus HFRS 1.7:1) along with reductions in the abundance of class Clostridia (HF 76.18 ± 1.9 versus HFRS 38.70 ± 7.3, *p* < 0.001) and the genera *Lactococcus* (*p* < 0.01), *Facklamia* (*p* < 0.05)*, Peptococcus* (*p* < 0.05)*, Dorea* (*p* < 0.05)*,* and *Rothia* (*p* < 0.05). The RS supplementation also normalized abundances of Bacteroidetes (*p* < 0.001)*,* particularly *Prevotella* (*p* < 0.001). The RS supplementation led to a significant increase in Actinobacteria (*p* < 0.001) abundances compared to both chow-fed control and HF-fed rats. Probiotics *Bifidobacterium* are Actinobacteria, and RS supplementation specifically increased abundances of genera *Bifidobacterium* (*p* < 0.01) and *Collinsella* (*p* < 0.01). The LEfSe showed that Actinobacteria, in particular *Bifidobacterium,* were particularly characteristic of the HFRS-fed rats (Figure 2). Overall profile cluster analyses and PCA determined a strong relationship between diet and microbiota profile composition. Analysis at all taxa levels determined significant differences among all three diet groups (Figure 2A,B), with the strongest differences between the HF and HFRS groups. Potato RS supplementation resulted in a microbiota composition more closely related to chow than HF (Figure 2A,B,C) and a specific increase in *Bifidobacterium spp*.

### 3.3. Potato RS Increases Fecal SCFA Content

The HF feeding led to a significant decrease in fecal acetate contents compared to the chow diet (chow 198.3 ± 12.8 versus HF 55.9 ± 5.9 nmol/5 g feces, *p* < 0.0001) and fecal propanoic acid contents (chow 47.3 ± 6.7 versus HF 6.2 ± 1.2 nmol/5 g feces, *p* < 0.01). The RS supplementation prevented an HF diet-driven decrease in acetate (HFRS 202.3 ± 26.1 nmol/5 g versus HF, *p* < 0.0001, versus chow, *p* = 0.77) and propionate (HFRS 51.2 ± 10.5 nmol/5 g versus HF, *p* < 0.001, versus chow, *p* = 0.77). Lastly, chow feces contained significantly more butyric acid than the HF animals (chow 52.3 ± 7.0 versus HF 6.6 ± 1.3, *p* < 0.01) and HFRS animals (chow 52.3 ± 7.0 versus HFRS 19.9 ± 4.4, *p* < 0.05) (Figure 2D).

### 3.4. Potato RS Attenuates Hypertrophy and Inflammation

**Figure 3:**
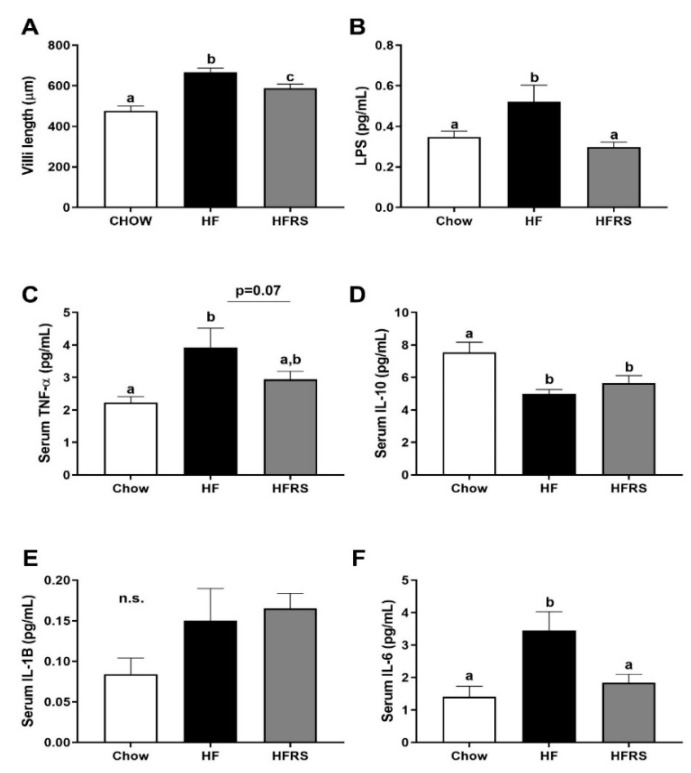
Potato RS attenuates GI hypertrophy and systemic inflammation. The HF diet increased villi length compared to chow control which were reduced in RS supplemented rats (**A**). The HF feeding led to a significant increase in pro-inflammatory LPS circulating levels, which was normalized by RS supplementation (**B**). Similarly, HF-diet driven increases in circulating TNFα (**C**) and IL-6 (**F**) were reduced in HFRS rats. Both HF and HFRS animals display a significant decrease in circulating IL-10 (**D**). There were no differences among groups in serum IL-1β (**E**). LPS = lipopolysaccharides, TNFα = tumor necrosis factor-a, IL-10 = interleukin 10, IL-6 = Interleukin 6, IL-1β = Interleukin 1 Beta, HF = high fat, HFRS = high-fat resistant starch, all significance determined at *p* < 0.05. *n* = 8 per group. Data are presented as the mean ± SEM; ^a, b, c^ different letters indicate statically significant differences among groups.

The HF feeding led to a significant increase in villus height compared to the chow control group (chow 476 ± 24.3 versus HF 667 ± 19.8 µm, *p* < 0.0001). The RS supplementation partially normalized villus height (Figure 3A). Villi high in the ileum of HRS-fed rats were significantly reduced compared to HF-fed animals (*p* < 0.05) but sill significantly higher than chow-fed rats (*p* < 0.01). There were no differences in goblet cell counts among the groups.

Serum LPS levels were elevated in the HF-fed rats compared to the chow control animals (Figure 3B) (chow 0.33 ± 0.07 versus HF 0.56 ± 0.08, *p* = 0.016). This was normalized by RS supplementation. Serum TNFα levels in HF animals were significantly elevated compared to the chow control (Figure 3C) (chow 2.22 ± 0.19 versus HF 3.93 ± 0.59 pg/mL, *p* < 0.01). This was partially reduced by RS supplementation (HF versus HFRS 2.95 ± 0.25, *p* = 0.1). Similarly, serum IL-6 levels were significantly increased in the HF animals (chow 1.41 ± 0.32 versus HF 3.45 ± 0.58 pg/mL, *p* < 0.01) and this was normalized in the HFRS rats (HF versus HFRS 1.84 ± 0.26 pg/mL, *p* < 0.05) (Figure 3F). Interestingly, serum IL-10 levels were significantly elevated in the chow animals compared to both HF (chow 7.6 ± 0.62 versus HF 5.0 ± 0.27 pg/mL, *p* < 0.01) and HFRS (chow versus HFRS 5.7 ± 0.46, *p* < 0.05) (Figure 3D). Serum IL-1B levels were not significantly different among groups (Figure 3E).

### 3.5. Potato RS Improves Glucose Tolerance

**Figure 4:**
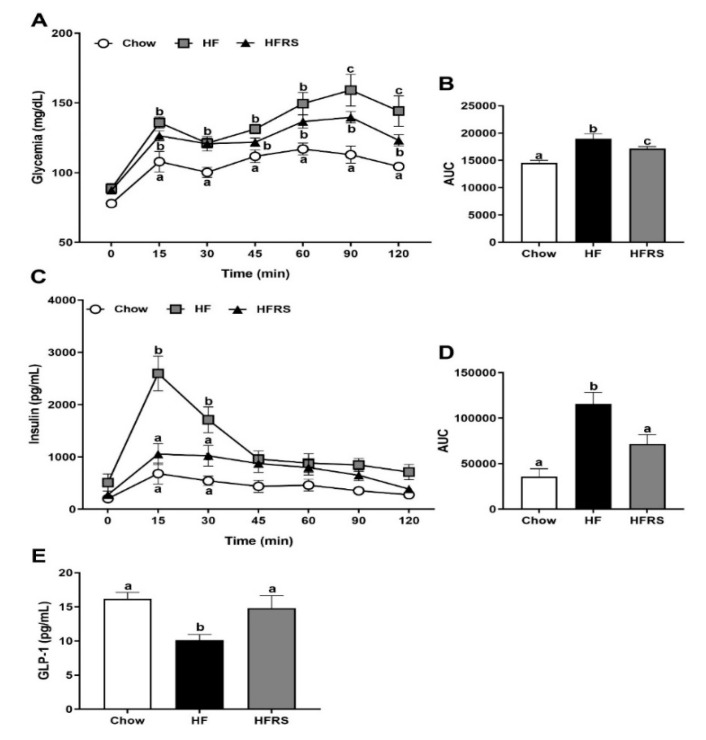
Potato RS improves glucose homeostasis. There was a significant increase in glycemia in all HF rats compared to chow animals in response to glucose, but HFRS rats recovered faster than HF; glycemia was significantly lower at 90 and 120 min in HFRS compared to HF (**A**). A significantly higher AUC was observed in HF than chow, which was partially improved in HFRS (**B**). Insulinemia was significantly higher in HF rats compared to both chow and HFRS animals at 15 and 30 min post-oral gavage (**C**,**D**). There was a significant decrease in circulating GLP-1 in HF-fed rats after 8 weeks on the diet compared to chow and HFRS rodents (**E**). OGTT = oral glucose tolerance test, AUC = area under the curve, HF = high fat, HFRS = high-fat resistant starch. Data are presented as the mean ± SEM; ^a, b, c^ different letters indicate statically significant (*p* < 0.05) differences among groups, *n* = 8 per group.

An OGTT was conducted after 7 weeks on the respective diets. There was a significant increase in baseline glycemia in both HF- and HFRS-fed rats compared to chow-fed controls (Figure 4A). Glycemia increased in all groups following an oral glucose challenge and staid elevated throughout the sampling period. The HF and HFRS glycemia was significantly higher than the chow control group at 15, 30, 45, and 60 min post challenge. The HFRS rats recovered faster than HF rats from this high glycemic episode, and the HFRS rats’ circulating glucose levels were significantly lower than HF-fed rats at 90 and 120 min post challenge (90 min HF 159.1 ± 11.4 versus HFRS 139.7 ± 4.1 mg/dL, *p* < 0.05 and 120 min HF 144.2 ± 11.0 versus HFRS 123.2 ± 4.0 mg/dL, *p* < 0.01) but stayed elevated compared to the chow control rats (90 min chow 112.9 ± 6.2 mg/dL versus HFRS, *p* < 0.001 and 120 min chow 104.4 ± 1.5 mg/dL versus HFRS, *p* < 0.05). The overall glycemic response (area under the curve, AUC) was significantly altered in HF-fed rats which was partially but not fully improved by RS supplementation (Figure 4A,B).

Insulin levels were increased in all groups in response to the glucose challenge. Insulinemia peaked at 15 min post gavage. Insulin levels in HF-fed rats were significantly higher than in the chow control group at 15 (chow 683 ± 201 versus HF 2597 ± 330 pg/mL, *p* < 0.0001) and 30 min (chow 542 ± 91 versus HF 1711 ± 247 pg/mL, *p* < 0.0001). The RS supplementation normalized glucose-induced insulin release; there was no significant differences in insulinemia between the HFRS rats and chow animals throughout the OGTT time course (Figure 4C,D).

Lastly, GLP-1 levels were measured in serum collected at sacrifice, and HF-fed rats displayed significantly reduced circulating GLP-1 levels compared to chow animals (chow 16.2 ± 0.9 versus HF 10.1 ± 0.8 pg/mL, *p* < 0.01); this was normalized by RS supplementation (Figure 4E).

### 3.6. Potato RS Prevents HF Diet-Driven Loss in CCK Satiety

**Figure 5:**
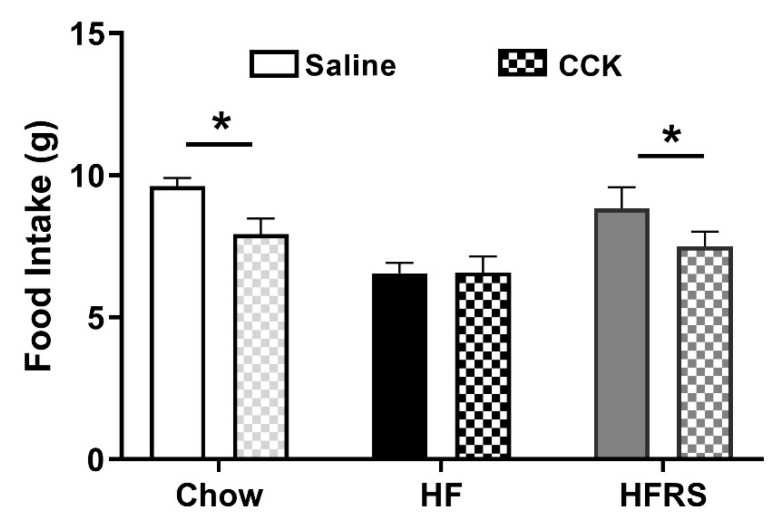
Potato RS prevents HF-driven loss in CCK sensitivity. Two hours post i.p. injection, chow and HFRS animals significantly decreased food intake when injected with CCK; compared to saline injection, HF rodents did not significantly decrease food intake with CCK injection. CCK = cholecystokinin, i.p., = intraperitoneal, HF = high fat, HFRS = high-fat resistant starch, * indicates significance determined as *p* < 0.05. *n* = 7–8 per group. Data are presented as the mean ± SEM.

A CCK-sensitivity test was conducted after 6 weeks on respective diets. After a 12 h fast, food intake was recorded for 2 h following no injection (control) or an i.p. injection of saline (400 µL) or CCK (0.22 nmol/kg). For all groups, there were no significant differences in food intake between control and saline injection conditions (data not shown). In chow-fed rats, CCK injections led to a significant reduction in food intake compared to the saline conditions (saline 9.6 ± 0.3 versus CCK 7.9 ± 0.6 g, *p* < 0.05). This CCK-induced satiety response was lost in HF-fed rats; animals consumed as much food when they administered CCK as when they received saline (saline 6.6 ± 0.4 versus CCK 6.6 ± 0.6 g, *p* = 0.97). RS supplementation prevented HF-diet driven loss in CCK-induced satiety; there was a significant reduction in intake following CCK administration compared to saline in HFRS rats (saline 8.8 ± 0.7 versus CCK 7.5 ± 0.5 g, *p* < 0.05) (Figure 5).

### 3.7. Potato RS Reduces NTS Microglia

**Figure 6:**
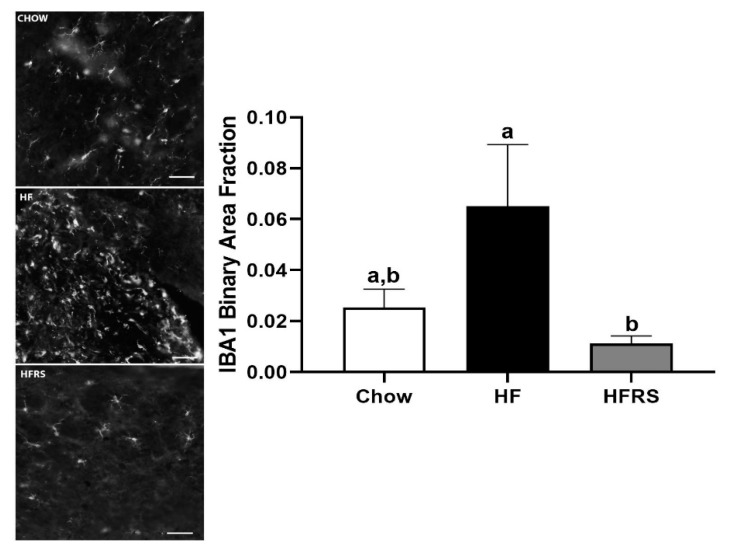
Fluorescent staining for IBA1 quantification showed a significant increase in positive staining in the NTS of HF-fed rats compared to HFRS animals (**right**). Representative images showing positive staining and morphology of microglia in chow, HF, and HFRS NTS sections (**left**). NTS = nucleus tractus solitarius, IBA1 = isolection B-alpha 1, HF = high fat, HFRS = high-fat resistant starch, a, b different letters indicate statically significant (*p* < 0.05) differences among groups, *n* = 6–7, scale bar = 50 µm.

We quantified IBA1-positive staining at the level of the medial NTS. The HF feeding led to a significant increase in IBA1-positive staining intensity in the NTS compared to HFRS (HF 0.065 ± 0.024 versus HFRS 0.011 ± 0.003, *p* < 0.05), but this did not reach significance when compared to chow controls (chow 0.025 ± 0.007 versus HF, *p* = 0.127) (Figure 6). Increased intensity of IBA1 revealed a seemingly increased microglia presence and/or activation in HF animals whereas RS supplementation attenuated this neuroinflammatory response (Figure 6).

### 3.8. Potato RS Prevents Vagal Remodeling

**Figure 7:**
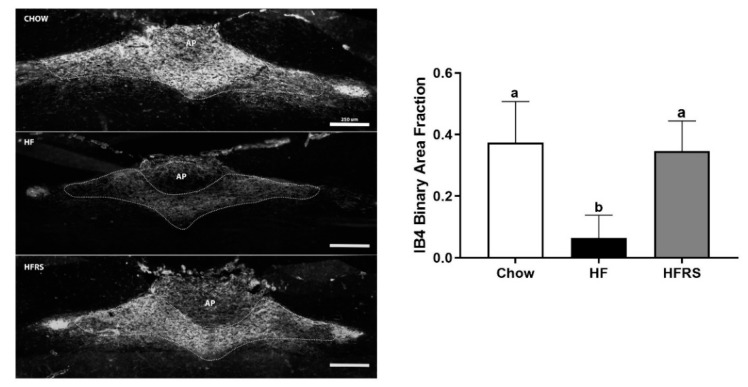
Potato RS prevented vagal remodeling. Representative NTS sections showing IB4 in chow, HF, and HFRS animals; the medial NTS at the level of the area postrema is outlined in white dashes (**right**), positive staining was quantified in the marked area (**left**). NTS = nucleus tractus solitarius, IB4 = isolectin B4, HF = high fat, HFRS = high-fat resistant starch. Data are presented as the mean ± SEM; ^a, b, c^ different letters indicate statically significant (*p* < 0.05) differences among groups, *n* = 8. Scale bar = 250 µm.

We quantified IB4-positive staining at the level of the medial NTS. The HF diet significantly reduced IB4 staining in the NTS compared to the chow control (chow 0.37 ± 0.05 versus HF 0.06 ± 0.09 binary area fraction, *p* < 0.0001). Potato RS prevented the diet-induced loss in c-fibers positive staining. There was significantly more IB4 positive fluorescence in the NTS of HFRS rats compared to HF-fed animals (HF versus HFRS 0.35 ± 0.04 binary area fraction, *p* < 0.001). The c-fiber innervation in the NTS was comparable in the chow- and HFRS-fed rats (Figure 7).

## 4. Discussion

Our study found that potato RS supplementation reduced weight gain and prevented HF-diet-induced hyperphagia. Expectedly, RS supplementation improved microbiota dysbiosis and increased fecal SCFA content. Compared to HF rodents, the HFRS rats exhibited lower levels of inflammation. Functionally, the HFRS rodents improved glucose homeostasis, and RS supplementation prevented HF diet-driven loss in CCK satiety. For the CCK signals, predominantly through activation of the vagus nerve, we found that potato RS prevented vagal remodeling and recruitment of immune cells at the level of the NTS, potentially preserving vagally mediated satiety signaling.

Potato RS supplementation successfully attenuated weight gain associated with a HF diet. Although, HFRS rodents gained significantly more weight than chow controls despite no overall significant difference in energy intake. While HFRS did consume more energy on a certain week, the difference in body weight may not be solely due to kcal intake. Other factors could include energy expenditure (which was not measured), microbiota composition and fecal energy harvest, and dietary macronutrient composition. Specifically, adiposity in rats has previously been shown to be proportional to dietary fat contents [43]. Additionally, HFRS rodents’ meal patterns may also have been different from the chow-fed rats, which can influence body weight [44,45].

Potato RS supplementation led to a marked improvement in diet-driven microbiota dysbiosis. The microbiota is the community of commensal, symbiotic, and pathogenic microorganisms that coexist in the human body [46]. In the GI microbiota alone, there are more than 10^14^ bacteria and over 400 bacterial species [46]—more than 10 times bacterial cells than human cells. Ninety-five percent of the gut microbiota is composed of two major phyla: Firmicutes and Bacteroidetes [6,9,47]. In our study, the HF diet increased the Firmicutes:Bacteriodetes ratio, a consistent finding in response to a HF diet [47]. Within Firmicutes, the classes Clostridia and Erysipelotrichia bloomed in the HF-fed rats, while the abundances of the Bacteroidales (order, Bacteriodetes) were significantly decreased compared to chow-fed animals, again consistent with previous findings [7,27]. These changes were associated with a significant decrease in SCFA content in the feces of HF-fed rats.

Around 100–200 mmol of SCFAs are produced daily in the human colon [48]. Diets higher in insoluble fibers promote SCFA production by colonic anaerobic bacteria [48]. These microbiota-accessible carbohydrates—fructooligosaccharides, cellulose, and resistant starches—are degraded by “primary degraders”, such as *Bifidobacterium* spp., *Bacteriodes* spp., and *Ruminococcus Bromii* [49], and broken down into propionate, acetate, and glucose. In our study, we found that the microbiota of potato RS-supplemented rats were significantly enriched in *Bifidobacterium* spp. and Bacteroidale*s*, the order of bacteria containing the previously mentioned *Bacteriodes* spp. Previous studies have found similar increases in *Bifidobacterium* in response to fiber and/or RS supplementation [50,51,52,53]. These changes were associated with higher levels of SCFAs in the distal gut, particularly acetate and propionate. Interestingly, propionate and acetate have been shown to play opposite roles in hepatic lipogenesis. Propionate appears to prevent liver lipid accrual [54], while acetate promotes it [55]. Increased acetic acid levels in HFRS rats may explain the lack of differences in liver lipid levels between HFRS and HF rodents. Critical in gut health, SCFAs promote mucus secretions, cell survival, and tight junction proteins’ integrity [56]. The HF-fed rats had significantly longer villi in the ileum than chow-fed animals. Increased villi length has been found with abnormal cell proliferation in GI disease states [57], supporting a decrease in gut function in HF-fed rats. The RS supplementation prevented HF diet-driven villi hypertrophy. Additionally, both acetate and propionate are activators of G-protein coupled receptors *ffar2* and *ffar3*, which increases peptide YY (PYY) and GLP-1 release when activated [58]. In our study, the HFRS rats displayed an increase in circulating GLP-1 compared to the HF-fed animals.

Improvements in gut function were associated with reduced systemic inflammation and improved glucose homeostasis in potato RS-supplemented rats. Diet-driven dysbiosis combined with impaired gut epithelial function allows for translocation of bacterial factors such as pro-inflammatory LPS to the circulation [13]. Chronic elevation in circulating LPS is sufficient to promote hyperphagia [15] and insulin resistance [23]. Notably, LPS promotes cytokines release [59]. In this study, RS supplementation decreased circulating serum levels of LPS and several pro-inflammatory cytokines compared to HF-fed rats. This effect may be mediated via SCFAs, as propionate supplementation has been found to decrease LPS-induced inflammatory response [60]. Chronic inflammation is a key triggering factor in the development of insulin resistance; pro-inflammatory cytokines can notably impair insulin receptor substrate-1 (IRS-1) signaling [61]. As previously reported [62], a HF diet led to an increase in serum TNF-α which was reduced in HFRS rats (*p* = 0.07). Similarly. The IL-6 circulating levels were elevated in the HF-fed rats which was normalized in the HFRS animals. The RS supplementation led to a decrease in the overall inflammatory tone which may explain why HFRS rats required less insulin to clear the same amount of glucose than the HF-fed rats. Increased circulating GLP-1 may also be linked to improved insulin sensitivity [63]. The GLP-1 receptor agonists have been found to decrease LPS-induced secretion of inflammatory cytokines such as TNF-α [64]. Positive effects of RS in glycemic control have been reported across species: RS supplementation in obese dogs is more effective in controlling glucose responses than soluble starches [65] and supplementation with RS has been shown to decrease fasting glucose, pro-inflammatory markers, cholesterols, waist circumferences, and percent body fat in humans with signs of metabolic syndrome [66].

Along with insulin resistance, bacterial inflammation also affects gut–brain signaling. Post-ingestive signals originating from the GI tract are relayed via the vagus nerve to the NTS to control meal size [67]. High-fat feeding alters gut–brain signaling resulting in overeating [68]. We previously found that chronic LPS administration is sufficient to impair vagally mediated satiety and increased intake [15], pointing towards a causal link between dysbiosis and diet-driven alteration in gut–brain communication. In addition to affecting vagal function, HF feeding leads to structural changes in vagal innervation. The vast majority of unmyelinated c-fibers at the level of the medial NTS level are of vagal origin [69] and can be labeled with IB4. The HF feeding leads to a reduction in NTS IB4 staining [7], potentially affecting vagal function. Bacterial inflammation may play a key role in diet-driven vagal remodeling; a decrease in IB4 staining is accompanied by an increase in microglia recruitment along the gut–brain axis [7] and antibiotic administration prevents dysbiosis, microglia recruitment, changes in vagal structure, and hyperphagia in HF-fed rats [19]. Data from this study support a role for the microbiota in driving vagal maladaptation. Potato RS supplementation prevented diet-driven dysbiosis and maintained vagal NTS innervation pattern and sensitivity to gut satiety peptide CCK. Reduced sensitivity of post-ingestive negative feedback may explain the maintenance of hyperphagia in HF but not HFRS rodents [4].

Impaired vagal signaling may play a role in altering insulin release in response to glucose. Our results are consistent with previous studies that have found that HF diets increased serum insulin concentration immediately following glucose stimulus [70]. Interestingly, HF diet rats who underwent vagotomy prior to glucose tolerance test do not exhibit a large release of serum insulin [70].

## 5. Conclusions

Targeting the microbiota in obesity maybe an effective non-invasive therapeutic approach. Microbiota modulation through resistant starch supplementation, specifically from raw potato starch, may prove to be an effective preventive and treatment strategy for obesity through the development of functional foods. Composition of the gut microbiota is ever changing in relation to diet composition and other environmental factors. In HF diet models, gut microbiota dysbiosis decreases intestinal barrier function and initiates inflammatory responses. Increased SCFA production as a result of RS supplementation may attenuate the effects of a HF diet by improving gut barrier function, reducing systemic LPS levels, and increasing GLP-1 levels. Functionally, potato RS supplementation prevented hyperinsulinemia and maintained glucose homeostasis compared to HF feeding alone. The RS supplementation also prevented diet-driven inflammation and remodeling of the gut–brain signaling, preserving vagally mediated satiety.

## Figures and Tables

**Table 1:** Macronutrient composition of chow, HF, and HFRS as percent grams and energy.

	CHOW		HF		HFRS	
	Gram %	kcal %	Gram %	kcal %	Gram %	kcal %
Fat	4.5	13.1	24	45	23	45
Protein	20	24.5	24	20	23	20
Carbohydrates	53.5	62.4	41	35	43	35
Sucrose	3.2	3.2	20.1	17	20.1	17
Fiber	6	0	5.8	0	5.8	0
RS	1.4	0	0.1	0	11.9	0
Energy Density (kcal/g)	3.4		4.7		4.6	

Note: Maltodextrin was added to the HFRS diet to match energy density. Diets prepared by Research Diets, Inc. HF = high-fat diet, HFRS = high-fat resistant starch, RS = resistant starch, kcal = calories.

## Supplementary Materials

The following are available online at https://www.mdpi.com/2072-6643/11/11/2710/s1, Table S1: Diet composition of HF and HFRS diets; Table S2: Resistant starch in raw potato starch, chow, HF, and HFRS diets; Figure S1: Microbiota abundance by taxonomic level.
