# Supplementary material for: Potato-Resistant Starch Supplementation Improves Microbiota Dysbiosis, Inflammation, and Gut–Brain Signaling in High Fat-Fed Rats
Source: Nutrients. 2019 Nov 8;11(11):2710. doi: 10.3390/nu11112710 (PMC6893629; doi:10.3390/nu11112710)
Supplement: Supplementary file 1 [file nutrients-11-02710-s001.pdf]

Supplemental Table S1. Diet composition of HF and HFRS diets[1]

Diet ingredient (g)	HF	HFRS
**Casein**	200	200
**L-Cystine**	3	3
**Corn Starch**	72.8	0
**Maltodextrin 10**	100	26
**Raw Potato Starch[2]**	0	172.8
**Sucrose**	172.8	172.8
**Cellulose**	50	50
**Soybean Oil**	25	25
**Lard**	177.5	177.5
**Mineral Mix S10026**	10	10
**Dicalcium Phosphate**	13	13
**Calcium Carbonate**	5.5	5.5
**Potassium Citrate, 1 H2O**	16.5	16.5
**Vitamin Mix V100001**	10	10
**Choline Bitartrate**	2	2
**FD&C Yellow Dye #5**	0	0
**FD&C Red Dye #40**	0.05	0
**FD&C Blue Dye #1**	0	0.05
**Total**	**858.15**	**884.15**

[1]Diets prepared by Research Diets, Inc. HF=high fat diet, HFRS=high fat resistant starch. [2] Potato starch was 60% resistant starch.

Supplemental Table S2. Resistant starch in raw potato starch, Chow, HF, and HFRS diets

	Potato Starch	Chow	HF	HFRS
% RS (g)	60	1.4	0.1	11.9

[1] HF, high fat; HFRS, high fat resistant starch; RS, resistant starch.

Online Supporting Material

Supplemental Figure S1. Microbiota Abundance by Taxonomic Level[1].

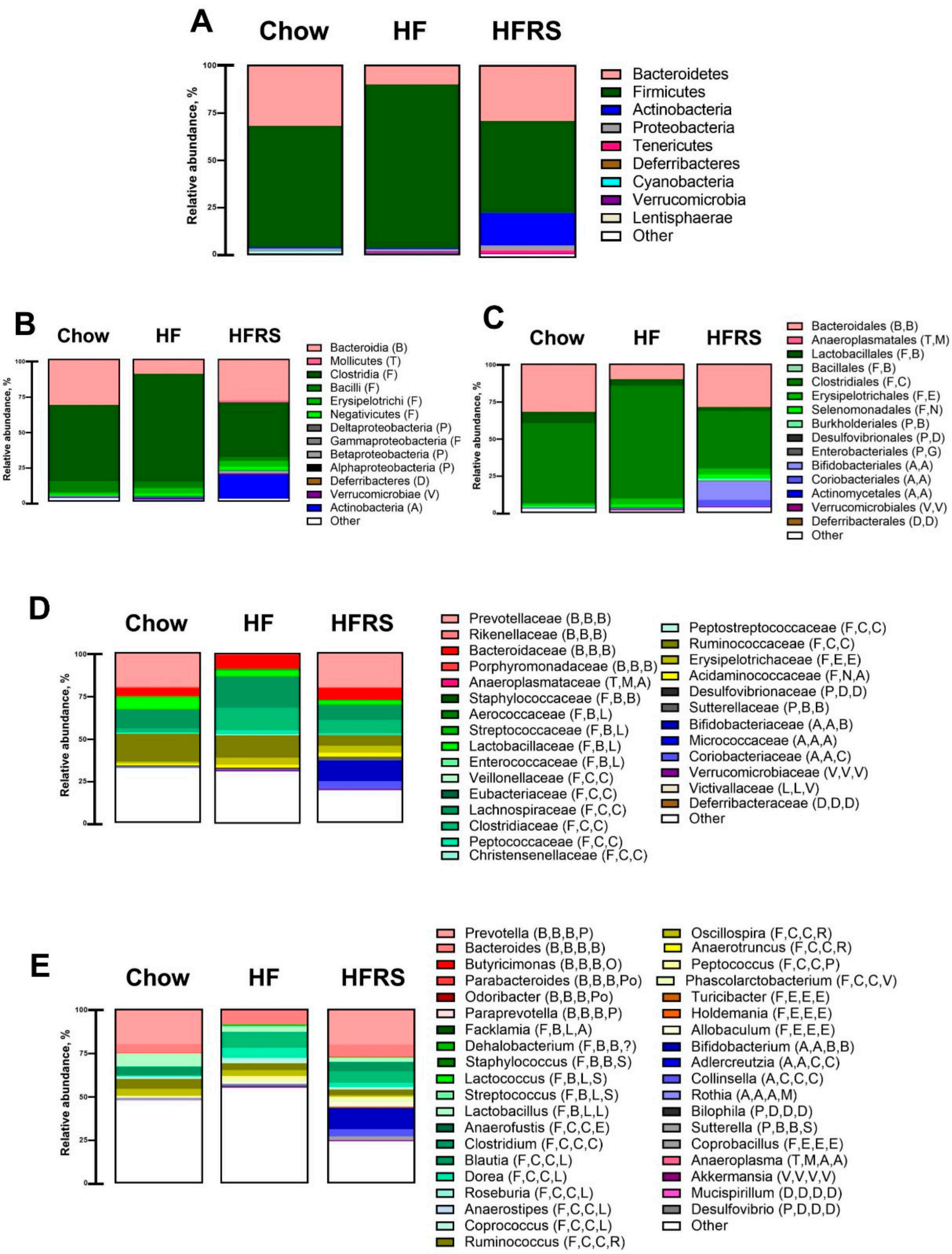

[1]Microbiota abundances for Chow, HF, and HFRS. **Phyla:** B: *Bacteroidetes*, F: *Firmicutes*, T: *Tenericutes*, P: *Proteobacteria*, A: *Actinobacteria*,V: *Verrucomicrobia* **Class:** B: *Bacilli*, B: *Bacteroidia*, C: *Clostridia*, E: *Erysipelotrichia*; M: *Mollicutes*, N: *Negativicutes*, D: *Deferribacteres*, A: *Actinobacteria*, V: *Verrucomicrobiae*, B: *Betaproteobacteria*. **Order:** B: *Bacteroidales*, C: *Clostridiales*, L: *Lactobacillales*, E: *Erysipelotrichiales*, B: *Burkholderiales*, B: *Bifidobacteriales*. **Family:** C: *Clostridiaceae*, L: *Lachnospiraceae*, R: *Ruminococcaceae*, L: *Lactobacillaceae*, E: *Erysipelotrichaceae*, D: *Desulfovibrionaceae*, B: *Bifidobacteriaceae*, V: *Verrucomicrobiaceae*, A: *Acidaminococcaceae*. Phyla level (A), Class (B), Order (C), Family (D), and Genera (E). HF=high fat, HFRS=high fat resistant starch, n=8.
